# Construction of a highly saturated Genetic Map for *Vitis* by Next-generation Restriction Site-associated DNA Sequencing

**DOI:** 10.1186/s12870-018-1575-z

**Published:** 2018-12-12

**Authors:** Junchi Zhu, Yinshan Guo, Kai Su, Zhendong Liu, Zhihua Ren, Kun Li, Xiuwu Guo

**Affiliations:** 1 College of Horticulture, Shenyang Agricultural University, Shenyang, 110866 People’s Republic of China; 2 Ministry of Education Key Laboratory of Protected Horticulture, Shenyang, 110866 People’s Republic of China

**Keywords:** Genetic map, *Vitis*, Restriction site-associated DNA sequencing, Linkage group, Single nucleotide polymorphism

## Abstract

**Background:**

High-saturate molecular linkage maps are an important tool in studies on plant molecular biology and assisted breeding. Development of a large set of single nucleotide polymorphisms (SNPs) via next-generation sequencing (NGS)-based methods, restriction-site associated DNA sequencing (RAD-seq), and the generation of a highly saturated genetic map help improve fine mapping of quantitative trait loci (QTL).

**Results:**

We generated a highly saturated genetic map to identify significant traits in two elite grape cultivars and 176 F_1_ plants. In total, 1,426,967 high-quality restriction site-associated DNA tags were detected; 51,365, 23,683, and 70,061 markers were assessed in 19 linkage groups (LGs) for the maternal, paternal, and integrated maps, respectively. Our map was highly saturated in terms of marker density and average “Gap ≤ 5 cM” percentage.

**Conclusions:**

In this study, RAD-seq of 176 F_1_ plants and their parents yielded 8,481,484 SNPs and 1,646,131 InDel markers, of which 65,229 and 4832, respectively, were used to construct a highly saturated genetic map for grapevine. This map is expected to facilitate genetic studies on grapevine, including an evaluation of grapevine and deciphering the genetic basis of economically and agronomically important traits. Our findings provide basic essential genetic data the grapevine genetic research community, which will lead to improvements in grapevine breeding.

**Electronic supplementary material:**

The online version of this article (10.1186/s12870-018-1575-z) contains supplementary material, which is available to authorized users.

## Background

Grapevine is a widely cultivated fruit crop worldwide, with high nutritional value. In 2016, 77 million tons were produced over a total area of 7 million ha (Food and Agriculture Organization). Several studies suggest that consumption of table grapes, grape products, and/or wine has many benefits for human health, and the requirement for high-quality grapes, including seedless and aromatic varieties, has increased considerably over the last several years [1–4].

Grapevine is a perennial woody plant species with a long juvenile period and is highly heterozygous; grapevine growth is negatively affected under various stressed conditions including natural disasters, disease, and pests. Identifying genes for desirable traits in grapevine cultivars via conventional cross-breeding techniques is challenging for cultivators and breeders. Therefore, alternative methods are necessary for large-scale production of cultivars with these traits. One method of achieving this is via construction of a map of molecular markers on a chromosome based on segregation data from a population resulting from a specific hybridization cross [5]. This approach has been used for numerous woody plants, for instance, to map quantitative trait loci (QTL) for plant quality traits and disease resistance [6].

Considerable progress had been made in the identification of molecular markers and the construction of molecular linkage maps in grapevine. The first molecular map for the 60 F_1_ progeny and the parental plants (F0) generation “Cayuga White” × “Aurore” was generated using 422 randomly amplified polymorphic DNAs (RAPDs), 16 restriction fragment length polymorphisms (RFLPs), and few isoenzyme markers [7]. Subsequent studies also used RAPDs, amplified fragment length polymorphisms (AFLPs), and sequence-related amplified polymorphisms (SRAPs) in F_1_ populations [8–12]. However, RAPDs, AFLPs, and SRAPs yield reportedly less stable results owing to uncontrollable experimental conditions [13] and have limited utility owing to their dominant pattern and low transferability. In contrast, simple sequence repeats (SSRs) have the advantages of co-dominance, high polymorphism, distribution throughout the genome, and good transferability with previously reported or annotated primer sequences [14–23]. (NCBI UniSTShttp://www.ncbi.nlm.nih.gov; Greek *Vitis* database, http://gvd.biology.uoc.gr/gvd/). Thus, reference genetic maps for the International Grape Genome Program (IGGP) have been constructed using 152 SSR markers and one polymorphic expressed sequence tag spanning 1728 cM [24]; 245 SSR markers spanning 1406.1 cM [14]; and 502 SSRs and 13 other types of PCR-based markers spanning 1647 cM [15]. However, in most cases, the total number of markers in the LGs is < 1000, with some lacking sequence-related information. In addition, there are some inconsistencies in LG number owing to the inefficiency and high cost of marker genotyping, which have prevented the fine mapping of target traits for breeding purposes. Therefore, to date, there are few high-density, high-quality genetic maps for grapevine, which encompass numerous molecular markers with detailed marker-related information.

The rapid development of NGS technologies and the publication of the grapevine reference genome sequence have assisted the identification of single nucleotide polymorphisms (SNPs), which have become the most widely used markers in genetic studies owing to their genomic abundance and stability [25, 26]. SNPs reportedly have revolutionary effects on high-quality genetic map construction [27, 28]. Several NGS-based methods have been used for simultaneous identification and scoring SNPs, including type IIB endonuclease restriction-site associated DNA (2b-RAD), double-digest (dd) RAD, genotyping-by-sequencing (GBS), specific length amplified fragment sequencing (SLAF-seq), and RAD sequencing (RAD-seq) [29–33]. RAD-seq is an NGS-based high-throughput sequencing technique, which simplifies the construction of highly multiplexed, low-representation libraries even in species with large genomes [31]. This method is a technology of reduced-representation genome sequencing (RRGS), with the advantages of simple operation, low experimental cost, and high throughput. RAD-seq is widely used in molecular biology, evolutionary genomics, population genetics, etc. For example, RAD markers were used to construct a high-density, high-quality genetic map in grapevine, which was subsequently applied for the detection of QTLs for sugar and acid production [34].

In this study, we used the F_1_ population derived from a cross between “Red Globe” *(V. vinifera* L*.*) and “Venus seedless” (*V. vinifera* × *V. labrusca.*). They were two table grapevine varieties that had significant differences in fruit size, ripening stage, disease resistance, fruit aroma, and number of seeds. Thus, the F1 generation is expected to segregate for labrusca aroma and disease resistance traits of these two elite grapevine cultivars. We performed RAD-seq to identify SNPs and insertion/deletion (InDel) markers to construct a highly saturated SNP-based molecular linkage map for grapevine, which can facilitate studies on grapevine ecology and evolution and facilitate the identification of QTLs for specific traits (grapevine aroma, white rot resistance, and downy mildew resistance), which will aid marker-assisted selection and accelerate genetic improvement of this important crop.

## Results

### Analysis of RAD-seq data for 176 F_1_ individuals and two parents

**Fig. 1:**
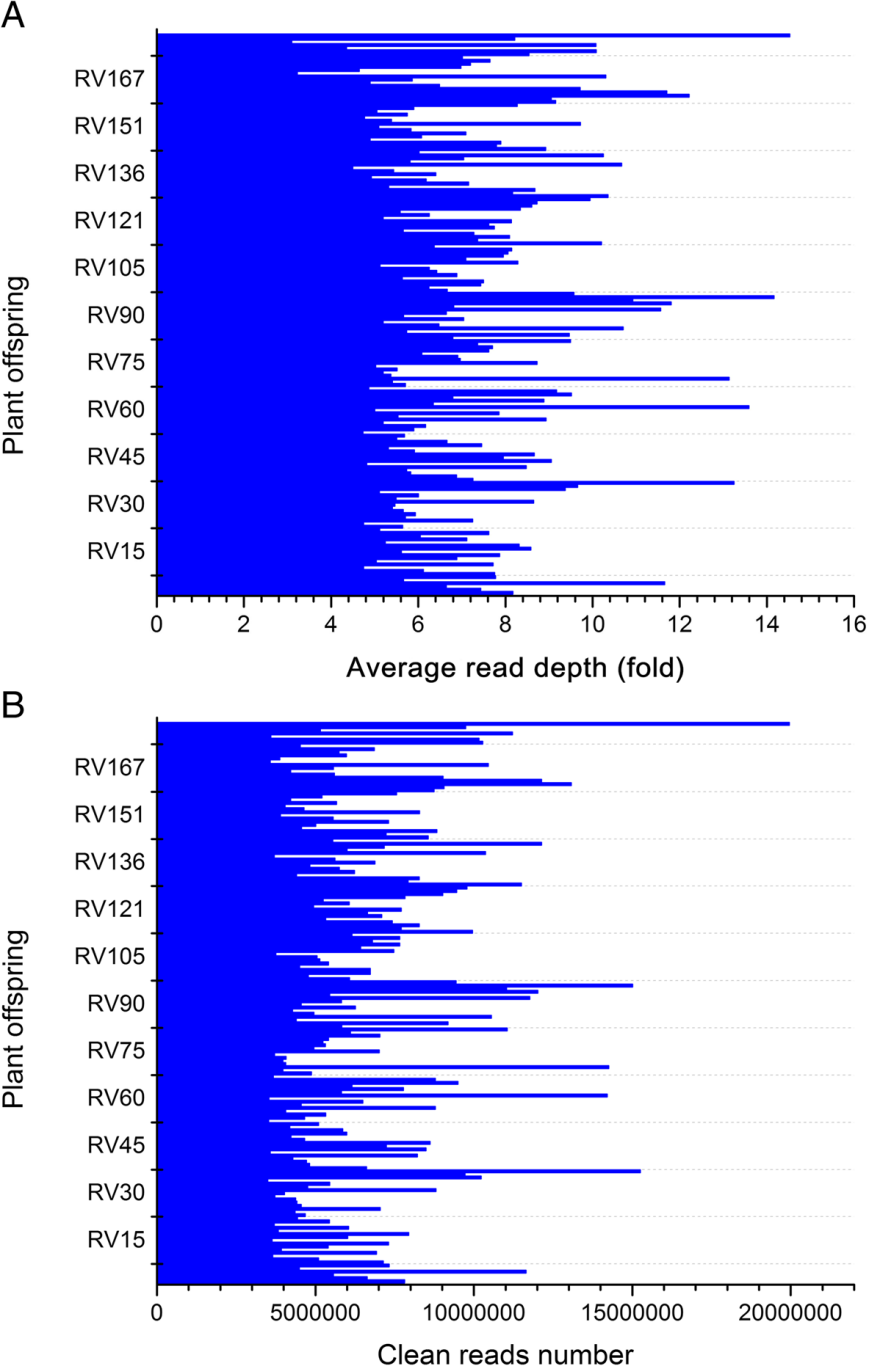
**a**, **b** Average read sequencing depth (fold) (**a**) and number of clean reads (**b**) expressed as genome equivalents of the 176 F_1_ individuals (shown in the X axis)

After treating the genomic DNA of F_1_ individuals and their parents with *Taq* I, samples were genotyped using high-throughput sequencing. After preprocessing, 388 Gb of raw data were obtained. To prevent sequencing errors, only reads showing < 5 bases with a Q score > 20 were further analyzed, yielding 206,411,693 clean reads ~ 150 bp in length; 94.39% were of a high quality, with quality scores of at least 30 (Q30, indicating a 0.1% chance of an error—i.e., 99.9% confidence). The guanine/cytosine content was 41.02% on average. A total of 1,426,967 RAD tags were detected; average sequencing depths were 52.7 for female parents “Red Globe,” 44.77 for male parents “Venus seedless,” and 7.25 for the progeny (Fig. 1a).

**Fig. 2:**
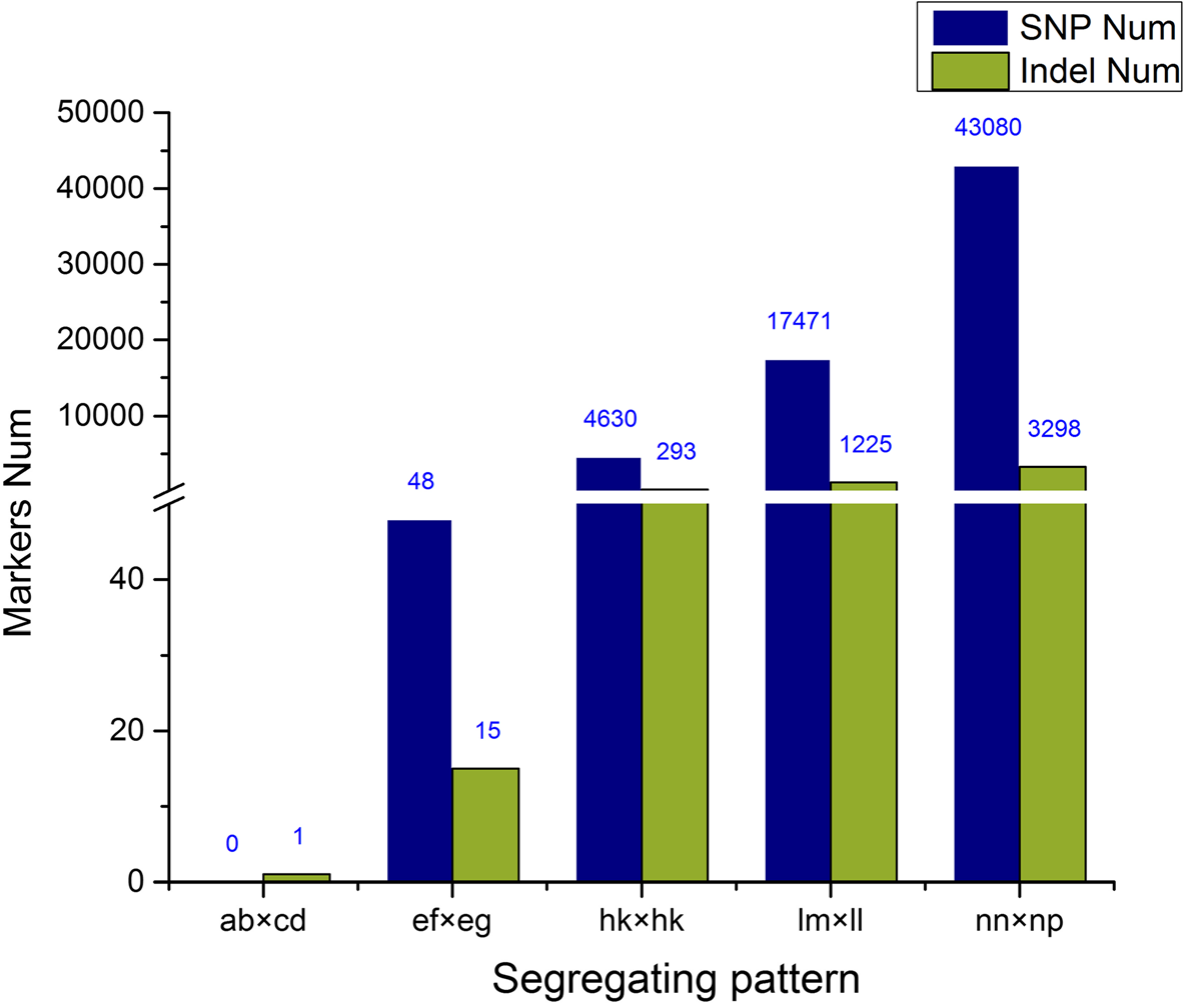
Number of markers in each of the five segregation patterns

Of these high-quality data, 85,503,839 clean reads were obtained for Red Globe; Venus seedless, 79,625,497 reads (Fig. 1b); per the criteria of segregation distortion (*P* < 0.05), 70,061 genome-wide DNA markers were used to construct a genetic map. The markers were classified into the following five segregation patterns: ab × cd, ef × eg, hk × hk, lm × ll, and nn × np (Fig. 2).

### Characteristics of the genetic maps

Mapped markers formed 19 linkage groups numbered in accordance with the chromosome number. LOD values ranged from 4 to 20 depending on the LG. There were 51,265 markers in the female map “Red Globe” (*V. vinifera* L.) and the total length was 3172.33 cM (Additional file 1: Figure S1). The length of each LG ranged from 125.56 cM for LG1 to 210.21 cM for LG9; mean length, 166.96 cM. LG1 contained the most markers (5325) with an average marker interval of 0.02 cM, whereas LG10 contained the fewest (1361) markers with an average marker interval of 0.11 cM. The “Gap ≤ 5 cM” percentage (gaps where the distance between adjacent markers was < 5 cM) for each LG ranged from 99.72% (LG8) to 100% (LG1, LG4, LG12, LG13, LG14, LG15, LG17, and LG19).

The map of the male parent “Venus seedless (*V. vinifera* × *V. labrusca*)” contained 23,683 markers spanning 3221.4 cM (Additional file 2: Figure S2). LG1(132.89 cM) and LG15 (210.12 cM) as the shortest and the longest linkage groups, respectively; mean length, 166.55 cM. LG1 contained 1488 markers with an average genetic interval of 0.09 cM, whereas LG15 contained 758 markers and an average genetic interval of 0.28 cM. The “Gap ≤ 5 cM” percentage for each LG ranged from 99.34% (LG3) to 100.00% (LG1, LG2, LG5, LG8, LG9, LG11, LG17, and LG19).

**Fig. 3:**
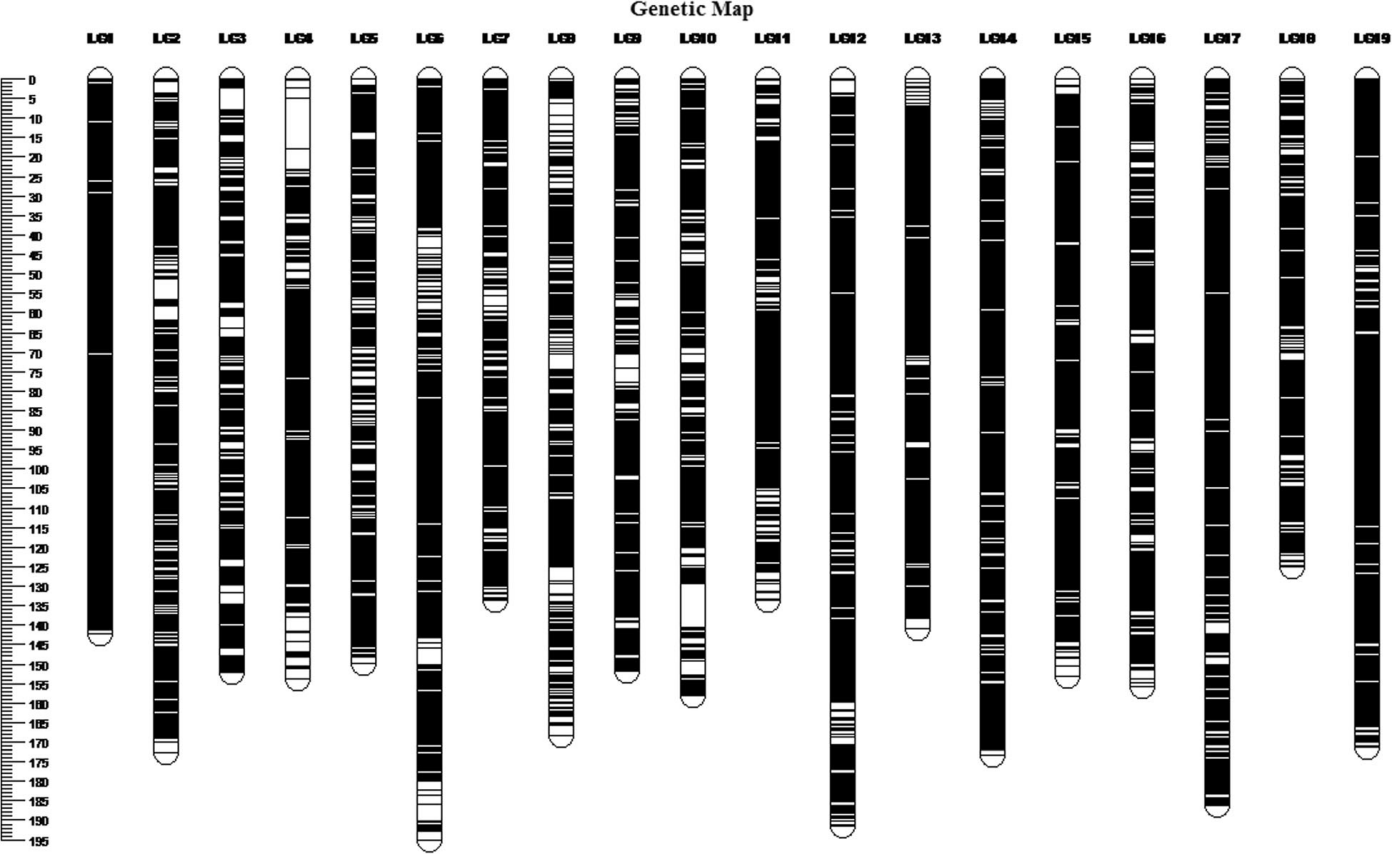
Genetic lengths and marker distribution in 19 linkage groups of the integrated map. Genetic distance is indicated by the vertical scale in centimorgans (cM). Black lines represent mapped markers. LG1–19 represent corresponding linkage groups ID

The integrated map contained a set of 70,061 markers spanning 3014.46 cM, with 3687 markers per LG on average and an average inter-marker distance of 0.05 cM (Fig. 3). The genetic length of LGs ranged from 125.17 cM (LG18) to 195.29 cM (LG6), with an average length of 158.66 cM. LG1contained the most markers (6564) spanning 142.42 cM with an average genetic interval of 0.02 cM, whereas LG3 spanned 152.42 cM and contained fewest markers (2031). The size and number of markers for each LG are described in Table 1. The average “Gap ≤ 5 cM” percentage was 99.99%. The ‘Gap > 5’ attribute was observed only in LG2, LG3, LG4, and LG9 (Table 2); two gaps > 10 cM were located in LG4 and one in LG10; however, they were only 12.91 cM (LG4) and 11.28 cM (LG10).

**Fig. 4:**
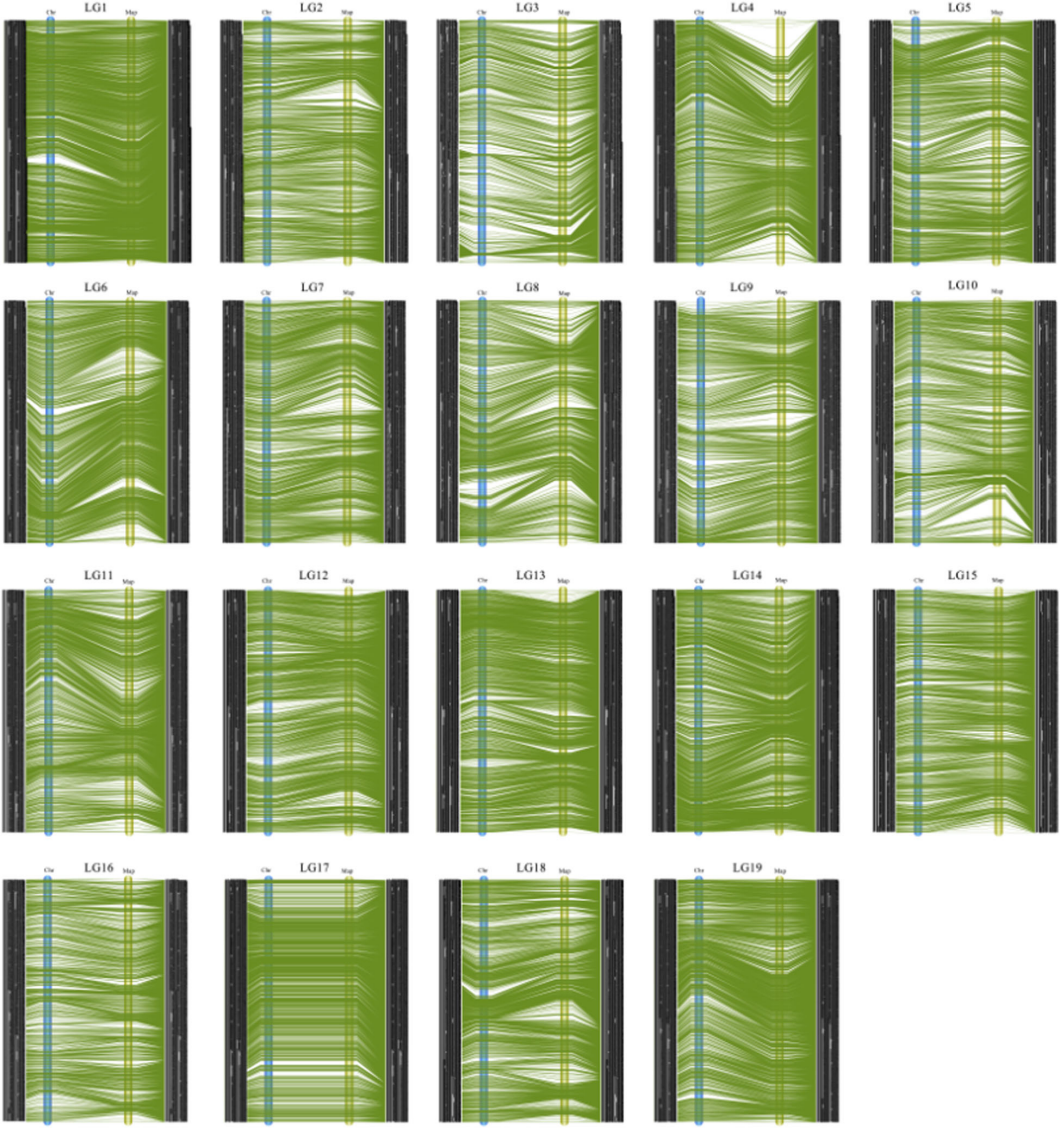
Collinear analysis of the consensus between genetic and physical maps

**Table 1:** The markers number and genetic distance of 19 linkage groups

Linkage groups (LG)	Total Marker			Total Distance(cM)		
	Red Globe	Venus seedless	Integrated map	Red Globe	Venus seedless	Integrated map
LG01	5325	1488	6564	125.56	132.89	142.42
LG02	1726	1448	2668	147.95	182.59	173.14
LG03	1519	602	2031	156.82	206.87	152.36
LG04	2132	1329	3188	190.56	159.21	154.12
LG05	1675	1891	3316	173.26	153.21	150.25
LG06	2406	1148	3250	157.52	186.94	195.29
LG07	1931	1349	3036	202.33	180.52	134.10
LG08	1432	1566	2697	134.66	172.21	168.81
LG09	1563	1074	2346	210.21	201.79	152.04
LG10	1361	1287	2405	155.47	192.23	158.34
LG11	2642	827	3302	185.32	136.76	134.14
LG12	2691	1236	3700	126.21	191.73	191.86
LG13	4410	906	5063	150.14	133.27	141.19
LG14	4059	1358	5090	174.71	172.64	173.74
LG15	2681	758	3355	149.87	210.12	153.42
LG16	1751	1136	2651	197.91	196.07	156.18
LG17	5027	973	5847	185.58	140.91	186.39
LG18	3206	1722	4453	155.95	140.42	125.17
LG19	3828	1585	5099	192.30	134.03	171.49
Total	51,365	23,683	70,061	3172.33	3224.40	3014.46

**Table 2:** The Average distance and Gaps ≤5 cM (Max gap) of 19 linkage groups

Linkage groups (LG)	Average Distance(cM)			Gaps≤5 cM (Max Gap)		
	Red Globe	Venus seedless	Integrated map	Red Globe	Venus seedless	Integrated map
LG01	0.02	0.09	0.02	100.00(0.61)	100.00% (3.72)	100.00% (0.96)
LG02	0.09	0.13	0.06	99.88(16.66)	100.00% (4.37)	99.96% (5.28)
LG03	0.1	0.34	0.08	99.87%(9.75)	99.34% (15.41)	99.95% (5.92)
LG04	0.09	0.12	0.05	100.00(4.77)	99.62(12.53)	99.94% (12.91)
LG05	0.1	0.08	0.05	99.88%(7.98)	100.00(4.86)	100.00% (2.01)
LG06	0.07	0.16	0.06	99.92(17.13)	99.83(17.24)	100.00% (4.30)
LG07	0.1	0.13	0.04	99.84(18.61)	99.70(14.15)	100.00% (2.55)
LG08	0.09	0.11	0.06	99.72%(8.32)	100.00(4.32)	100.00% (4.06)
LG09	0.13	0.19	0.06	99.94(20.88)	100.00(3.84)	100.00% (3.74)
LG10	0.11	0.15	0.07	99.85%(8.17)	99.77(11.96)	99.96% (11.28)
LG11	0.07	0.17	0.04	99.96%(6.12)	100.00(3.69)	100.00% (1.93)
LG12	0.05	0.16	0.05	100.00(1.39)	99.84(11.03)	100.00% (3.63)
LG13	0.03	0.15	0.03	100.00(1.71)	99.89(5.53)	100.00% (2.62)
LG14	0.04	0.13	0.03	100.00(2.08)	99.85(6.53)	100.00% (1.40)
LG15	0.06	0.28	0.05	100.00(4.70)	99.87(5.90)	100.00% (2.55)
LG16	0.11	0.17	0.06	99.94%(6.20)	99.91(7.93)	100.00% (2.44)
LG17	0.04	0.14	0.03	100.00(3.21)	100.00(4.97)	100.00% (3.06)
LG18	0.05	0.08	0.03	99.94%(6.92)	99.83(5.44)	100.00% (1.99)
LG19	0.05	0.08	0.03	100.00(1.63)	100.00(4.19)	100.00% (1.29)
Average	0.07	0.15	0.05	99.93%	99.87%	99.99%

‘Gaps≤5 cM’ indicated the percentages of gaps in which the distance between adjacent markers was smaller than 5 cM

**Fig. 5:**
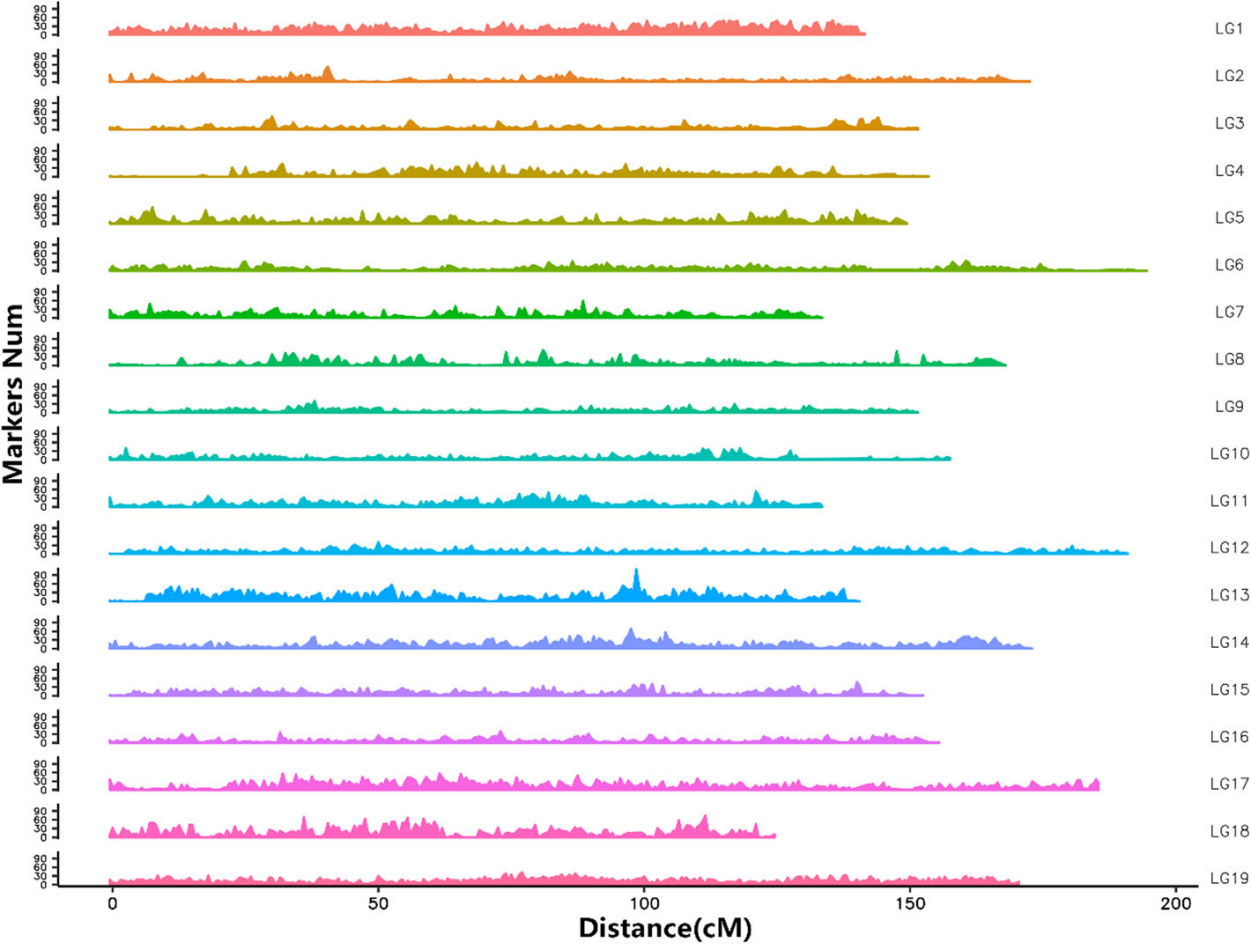
Marker density of integrated map.X-axis: physical position on 19 linkage groups. Y-axis: markers number per LG. The marker density on the genome was calculated by sliding windows using window size of 0.5 cM

**Fig. 6:**
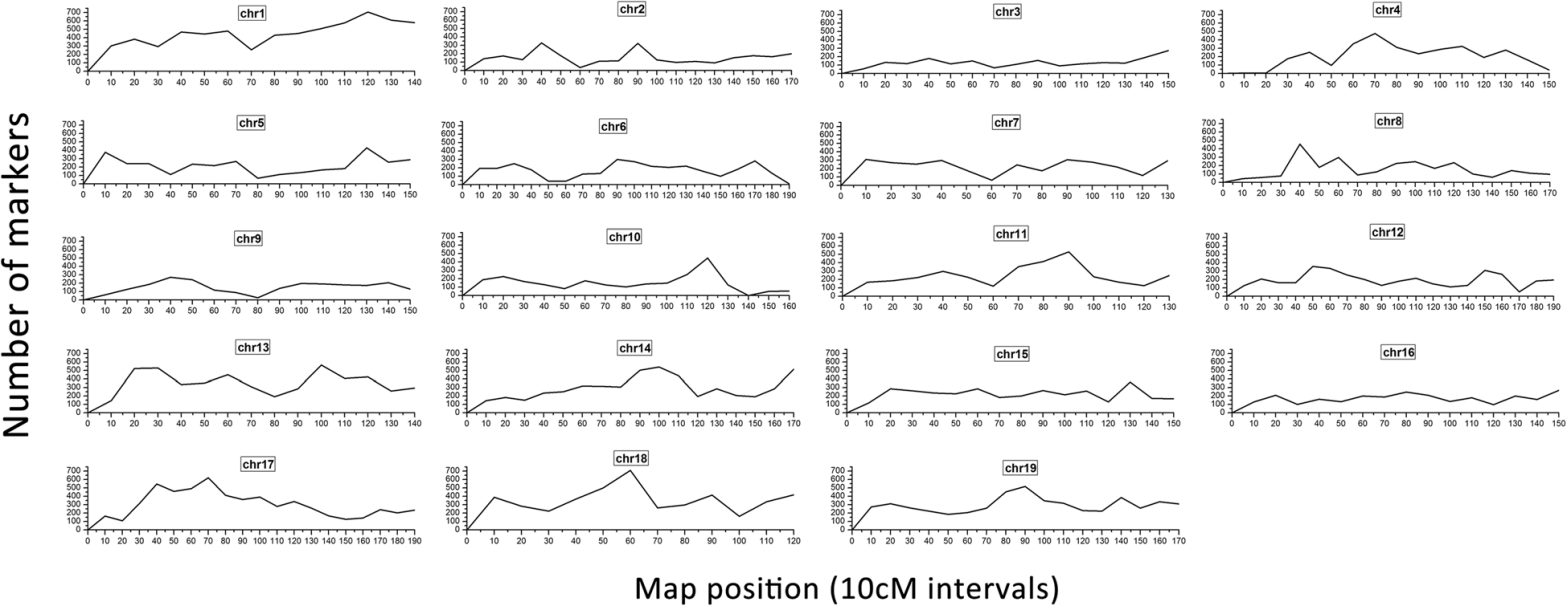
Distribution of marker density across the chromosome. The x-axis represents the 10 cM map interval and the y-axis represents the number of RAD markers present in the interval

### Comparative analysis of high-saturated linkage maps

The correlation between genetic and physical positions on a linkage map defines its quality [35].

To compare genetic and physical maps, we investigated the locations of all 65,229 SNP markers on the reference grapevine genome (Fig. 4). A high degree of collinearity was observed between genetic and physical distances of all SNP markers in the 19 LGs. All consecutive curves generated from the 19 LGs indicated that the reference genome was sufficiently encompassed with SNP markers positioned accurately within each LG. Most parts of these curves showed a declining trend, suggesting that their genetic and physical positions followed the same order.

To better describe the marker densities across the chromosome, we considered a sliding-window interval of 0.5cM and 10 cM across chromosome 19, respectively (Figs. 5 and 6).Physical coverage represents the proportion of chromosome length encompassed by all markers in the reference genome. In the 19 LGs, physical coverage ranged from 99.17% (LG9) to 99.98% (LG13), with an average of 99.83% (Table 3), indicating that most markers showed a good linear agreement between physical and genetic maps on the basic framework. The average Spearman correlation coefficient between the genetic and physical positions was 0.99, suggesting that the LGs exhibit high levels of genetic collinearity.

**Table 3:** Description on correlation coefficients between the genetic and physical positions of each linkage group on the integrated map

LG ID	Spearman	Physical Coverage	cM/Mb
Chr1	1	99.91%	6.19
Chr2	0.99	99.75%	9.24
Chr3	0.99	99.70%	7.9
Chr4	0.99	99.90%	6.46
Chr5	0.99	99.66%	6.03
Chr6	0.99	99.96%	9.08
Chr7	0.99	99.93%	6.38
Chr8	0.99	99.98%	7.54
Chr9	0.99	99.17%	6.66
Chr10	0.99	99.69%	8.76
Chr11	0.99	99.82%	6.78
Chr12	0.99	99.87%	8.46
Chr13	0.99	99.98%	5.79
Chr14	0.99	99.93%	5.74
Chr15	0.99	99.85%	7.57
Chr16	0.99	99.89%	7.09
Chr17	1	99.95%	10.88
Chr18	0.99	99.86%	4.27
Chr19	1	99.97%	7.14

cM/Mb: Genetic distance between markers reflected in the distance of each Mb in the genome

Heat maps can indicate the recombination frequency between markers within one single LG (Additional file 3: Figure S3); they can hence be used to identify potential markers ordering errors, pair-wise recombination occurring primarily owing to hotspot regions for genomic recombination and sequencing-related genotyping errors to optimize the genetic map. In general, most LGs yielded a good performance.

## Discussion

Genetic maps have long been used as a tool to improve grapevine cultivation and are indispensable for studies aimed at elucidating the genetic architecture of quantitative traits. Construction of a high saturated genetic map of grape is valuable for breeders because it potentially facilitates the identification of genomic regions with characteristics of agronomic interest [36].

Similar to other organisms, numerous SNPs have been used to characterize grapevine genomes [37–40] and construct high-density genetic maps [41–43]. A genetic map for *V. vinifera* was previously constructed with 994 markers (mostly consisting of 483 SNPs) spanning 1245 cM [41]. A consensus map for a grapevine cultivar (*V. vinifera* L.) derived from three crosses generated on the basis of 283 SSRs and 501 SNP-based markers was also added to the IGGP [42]. Although SNPs are presumed to be more numerous and genetically stable than other marker types, this is difficult for large scale detection.

An essential step in high-density map construction is scoring tens to hundreds of thousands of stable and accurate molecular markers in a cost-efficient manner. The continuously decreasing cost of NGS has resulted in the development of several NGS-based methods for SNP identification. One of these approaches is RAD-seq, which uses rare-cutter restriction enzymes (6- to 8-bp recognition site) for sequencing short DNA fragments surrounding a particular recognition site throughout the genome [33]. This method is adapted from the RAD tag marker technique for NGS platforms [44–46]. Several modifications of the original RAD-seq protocol have been reported, including 2b-RAD-seq methods [47], ddRAD-seq [30], and GBS [31]. For instance, in GBS, a frequent cutter enzyme is used to generate low-representation libraries prior to sequencing [31], although this leads to an increased rate of missing data, which is a major limitation of imputation programs [48, 49]. SLAF-seq was recently developed as a simplified sequencing technique potentially useful for large-scale screening of SNPs [32]. To date, several high-density genetic maps for grapevine have been constructed using NGS technology: RAD sequencing yielded 1646 SNP markers spanning 1917.3 cM from 100 F_1_ progeny and their parents [29] and 1826 SNP-based markers from 249 individuals and their parents [34]. GBS was used to construct genetic maps for *Vitis rupestris* “B38” (1146 SNPs) and “Chardonnay” (1215 SNPs) spanning 1645 and 1967 cM, respectively [50]. SLAF-seq of 149 F_1_ plants and their parents identified 7199 polymorphic markers in a map spanning 1929.13 cM [51]. This method also yielded 10,042 SNPs spanning 1969.95 cM from an analysis of 130 individuals and their parents [52].

In this study, RAD-seq of 176 F_1_ plants and their parents yielded 8,481,484 SNPs and 1,646,131 InDel markers, of which 65,229 and 4832, respectively, were used to construct a highly saturated genetic map for grapevine, spanning 3014 cM with an average coverage of 99.83% in 19 LGs. The average “Gap ≤ 5 cM” percentage of 99.99% indicated good uniform coverage, whereas the density of the linkage maps was highly saturated. Despite these advantages, the integrated map had two large gaps over 10 cM. The markers flanking these two gaps (LG04 and LG10) were aligned to the reference genome by BLAST. The flanking markers were physically located 16 and 32 kb apart, respectively. Recombination hotspots may be responsible for these results.

We developed 51,365, 23,683, and 70,061 markers for 19 linkage groups (LGs) for the maternal, paternal, and integrated maps, respectively. The genetic map requires molecular markers to display linear correlations with the chromosomes. Linkage maps were constructed as described previously [53], the Multipoint maximum likelihood method was enhanced by determining the degree of support of the possible conformational position of each molecule marker. For example, in LG1, marker chr1_320228, chr1_320235, and chr1_337168, these three markers were located at the same genetic position in close physical proximity. Subsequent studies may yield the location of the QTL in this region. We can increase the mapping population and recalculate the location of these markers for local fine mapping.

This genetic map of a cross with a complex parentage [(*V. vinifera*) × (*V. vinifera* × *V. labrusca*)] has the highest saturated compared to those constructed thus far for grapevine; moreover, it provides genomic tools to improve table grapevine cultivars; most characteristics of agronomic and economic importance in grape are quantitative traits, and it is very important in grape breeding to locate quantitative trait loci (QTLs) and estimate their effects. This genetic map successfully lays the foundation of fine mapping, marker-assisted selection, and cloning of QTLs.

Since the traits of hybrid offspring are inherited from the parents, parental selection is critical in constructing genetic maps. In this study, “Red Globe” (*V. vinifera* L.) was used as the maternal parent. This cultivar is one of the world’s most important table grape varieties and accounts for over 20% of the total grape cultivation area in China. It is characterized by large clusters and a large grape size, low acid content, firm flesh, and high productivity. It is also late-ripening and is well-preserved during storage and transportation. However, Red Globe shows poor cold tolerance and resistance to pathogens such as elsinoe anthracnose and downy mildew [54]. The paternal parent was “Venus seedless (*V. vinifera* × *V. labrusca*),” which is also extensively cultivated in China. It has a labrusca flavor, is seedless and highly resistant to disease, exhibits early ripening and strong growth [55]. Thus, the F_1_ generation is expected to segregate for the favorable traits of these two elite grapevine cultivars.

## Conclusion

In this study, RAD-seq of 176 F_1_ plants and their parents yielded 8,481,484 SNPs and 1,646,131 InDel markers, of which 65,229 and 4832, respectively, were used to construct a highly saturated genetic map for grapevine, spanning 3014 cM with an average coverage of 99.83% in 19 LGs. The average “Gap ≤ 5 cM” percentage of 99.99% indicated good uniform coverage, whereas the density of the linkage maps was highly saturated. This genetic map contains the largest molecular marker number of the grape maps so far reported. The genetic map will facilitate the QTL mapping of important grapevine traits in the future.

## Methods

### Mapping population and DNA isolation

An F_1_ grape hybrid of 176 individuals derived from a cross between “Red Globe” *(V. vinifera* L*.*) and “Venus seedless” (*V. vinifera* × *V. labrusca.*) was generated in May 2009. Stratification was performed between October 2009 and February 2010. Hybrid seeds were sown in a greenhouse in March 2010. A total of 531 crops were randomly harvested, of which 176 of them and each parent were used as the mapping population. The seedlings of the mapping population were sewn in batches in the vineyard of Shenyang Agriculture University, Liaoning Province, P. R. China (E123°24′, N41°50′) from April to June 2010.

Healthy young leaves (second or third leaves from the apex and less than 1 cm^2^) were harvested from both parents and each individual progeny plant (F_1_ generation). Samples immediately frozen in liquid nitrogen and quickly in store a − 80 °C refrigerator. Genomic DNA was extracted using the improved CTAB method [56]. Extracted DNA samples were treated with RNase A to eliminate residual RNA**.** DNA concentration and quality were evaluated using a NanoDrop 2000 spectrophotometer (Thermo Fisher Scientific, Waltham, MA, USA), finally the concentration and volume of each DNA sample was 500 ng·μl^− 1^ and 50 μl, the extracted DNA samples were electrophoresis on a 0.8% agarose gel. The DNA was diluted to a final concentration of 2.5 ng·μl^− 1^ for use in subsequent polymerase chain reactions (PCR).

### Library construction

RAD-seq libraries were constructed as described previously [33], with a few modifications. Briefly, genomic DNA (0.1–1 μg from either sample) was incubated for 5 min at 37 °C with 20 U of *Taq* I restriction endonuclease (New England Biolabs, Ipswich, MA, USA) in a 50-μl reaction mixture [57–59]. Individually barcoded P1 adapters were ligated to the *Taq* I restriction site for each sample. Thereafter, samples were pooled in proportional amounts for shearing to an average size of 500 bp with a Bioruptor (Diagenode, Liège, Belgium). Sequencing libraries were constructed a total of 24 samples per library. Libraries were size-selected for 450- to 550-bp fragments on a 2% agarose gel. Libraries were blunt end-repaired, and a 3′-adenine overhang was added to each fragment. We added a P2 adapter containing unique Illumina barcodes (San Diego, CA, USA) to each library. Libraries were amplified by via PCR, under the following conditions: 16 cycles (98 °C for 2 min; 16 cycles at 98 °C for 30 s, 60 °C for 30 s, and 72 °C for 15 s; and 72 °C for 5 min) with Phusion high-fidelity DNA polymerase (New England Biolabs) and column-purified. Samples were sequenced using a HiSeq 2500 system (Illumina) using 150-bp paired-end reads.

### In silico analysis

Quality trimming is an essential step to generate high-confidence variant calls. Raw reads were assigned to individual samples in accordance with their nucleotide barcode, using Axe package [60]. Raw reads were processed to obtain high-quality clean reads in accordance with three stringent filtering criteria: 1) elimination of reads with ≥ 10% unidentified nucleotides (N); 2) elimination of reads with > 50% bases having Phred quality scores ≤20; and 3) elimination of reads aligned to the barcode adapter. To identify SNPs, the Burrows-Wheeler Aligner (BWA) was used to align clean reads from each sample against the 12X.0 *Vitis vinifera* reference genome PN40024 (https://www.ncbi.nlm.nih.gov/assembly/GCF_000003745.3/) with the setting of “mem 4−k 32−M,” where −k is the minimum seed length and − M is an option used to mark shorter split alignment hits as secondary alignments [61]. The markers from a chromosome were used to construct the corresponding LG and the marker order were determined based upon the recombination fractions in the F1 population. Variant calling was performed for all samples, using the Genome Analysis Toolkit (GATK) Unified Genotyper (Broad Institute, Cambridge, MA, USA). Variants were filtered using standard hard filtering parameters in accordance with the GATK Best Practices pipeline. More precisely, SNPs and InDels were obtained on the basis of a mapping quality > 37 and quality depth > 24. Lastly, variants with > 70% call rate and sequence depth > 2-fold were used to construct a linkage map.

### Linkage map construction

Variants filtered by quality, as described above, were genotyped in accordance with their heterozygous parents into eight segregation types. After filtering those with no polymorphisms between parents or partial separation based on a *P* value < 0.01, markers with homozygous parents were used to construct a genetic linkage map for the F_1_ generation. Genetic marker data were scored in accordance with the criteria of JoinMap v.5.0 with a smooth algorithm. All statistical analyses described below were performed with a cross-pollinating-type population, using the same software designed to analyze data for the F_1_ outbreeding population containing various genotype configurations. Pairwise analyses were performed; markers were sorted into LGs at a minimum logarithm of odds (LOD) score of 4.0 and modified via genome location. The maximum recombination value was 0.3. Independence LOD scores were used as the grouping parameter, with the maximum likelihood (ML) mapping algorithm. Print map: each round only. The “locus genotype frequency” function was used to calculate chi-square values for each marker to test for the expected Mendelian segregation ratio. Markers deviating significantly from the expected ratio (*P* < 0.05) were excluded. Linkage distances were estimated for each LG, assuming the Kosambi mapping function [62]. The smooth algorithm was used to detect low-quality genotypes and impute the missing value. A consensus map was constructed using the “Join-combine groups for map integration” command.

## Additional files

Additional file 1: **Figure S1.** Genetic map of the male parent ‘Venus seedless (*V. vinifera* × *V. labrusca*)’. Genetic distance is centimorgans (cM) Kosambi. Black lines represent mapped markers. LG1–19 represent corresponding linkage groups ID. (DOCX 127 kb)

Additional file 2: **Figure S2.** Genetic map of the female parent ‘Red Globe’ (*V. vinifera* L.). Genetic distance is centimorgans (cM) Kosambi. Black lines represent mapped markers. LG1–19 represent corresponding linkage groups ID. (DOCX 89 kb)

Additional file 3: ** Figure S3.** Heat map of the genetic linkage map. (ZIP 3270 kb)

Additional file 4: **Table S1.** Markers used for mapping. (XLS 3199 kb)

## Abbreviations

CTAB: cetyltrimethylammonium bromide
IGGP: international grape genome program
LOD: logarithm of odds
NGS: next-generation sequencing
QTL: quantitative trait loci
RAD-seq: restriction-site associated DNA sequencing
RRGS: reduced-representation genome sequencing
SNPs: single nucleotide polymorphisms
