# Supplementary material for: Construction of a highly saturated Genetic Map for Vitis by Next-generation Restriction Site-associated DNA Sequencing
Source: BMC Plant Biol. 2018 Dec 12;18:347. doi: 10.1186/s12870-018-1575-z (PMC6291968; doi:10.1186/s12870-018-1575-z)

**Figure S1.** Genetic map lengths and marker distribution in 19 linkage groups of the male parent. Genetic distance is indicated by the vertical scale in centimorgans (cM). Black lines represent mapped markers. LG1-19 represent corresponding linkage groups ID.


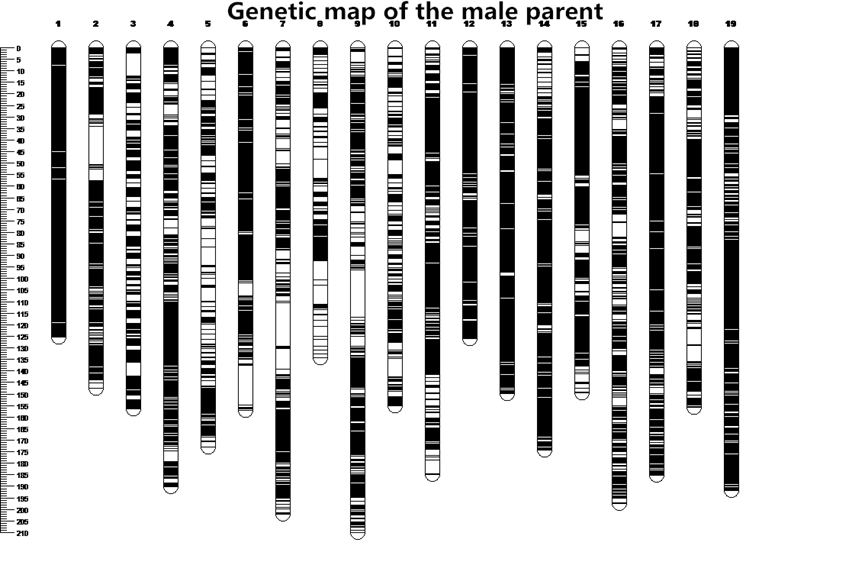

Supplement: Supplementary file 1 — Figure S1. Genetic map of the male parent ‘Venus seedless (V. vinifera × V. labrusca)’. Genetic distance is centimorgans (cM) Kosambi. Black lines represent mapped markers. LG1–19 represent corresponding linkage groups ID. (DOCX 127 kb) [file 12870_2018_1575_MOESM1_ESM.docx]
