# Supplementary material for: Construction of a highly saturated Genetic Map for Vitis by Next-generation Restriction Site-associated DNA Sequencing
Source: BMC Plant Biol. 2018 Dec 12;18:347. doi: 10.1186/s12870-018-1575-z (PMC6291968; doi:10.1186/s12870-018-1575-z)

**Figure S2.** Genetic map lengths and marker distribution in 19 linkage groups of the female parent. Genetic distance is indicated by the vertical scale in centimorgans (cM). Black lines represent mapped markers. LG1-19 represent corresponding linkage groups ID.


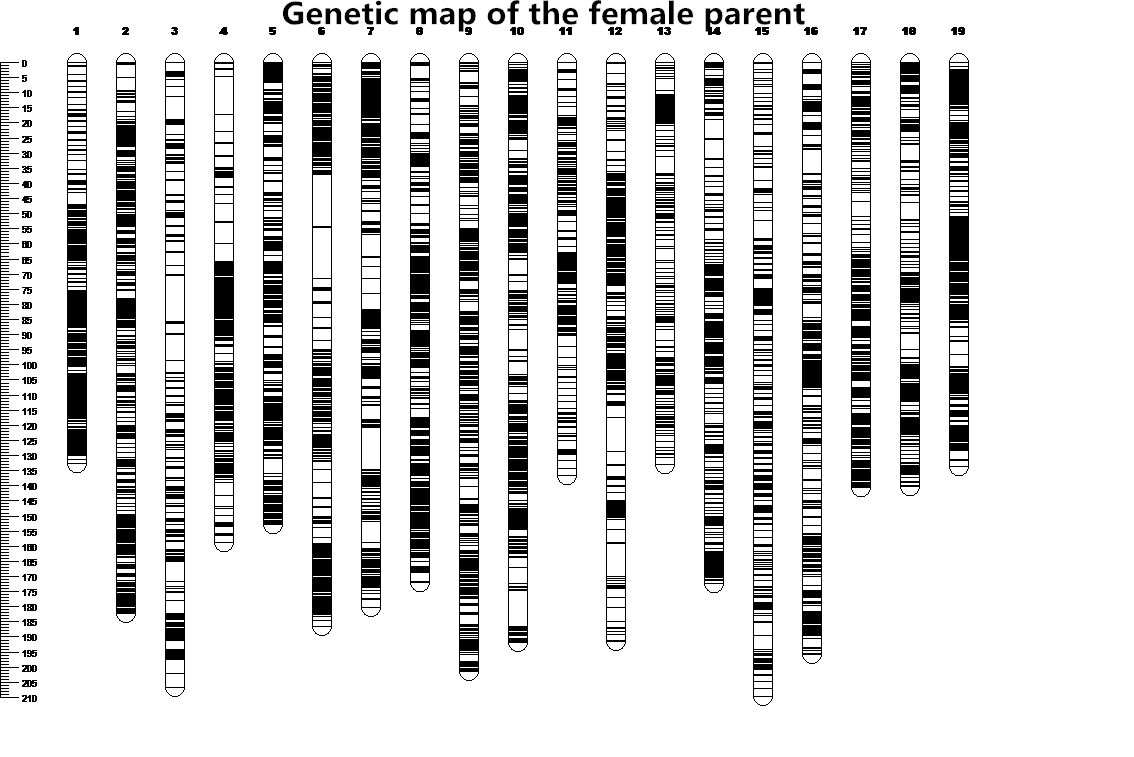

Supplement: Supplementary file 2 — Figure S2. Genetic map of the female parent ‘Red Globe’ (V. vinifera L.). Genetic distance is centimorgans (cM) Kosambi. Black lines represent mapped markers. LG1–19 represent corresponding linkage groups ID. (DOCX 89 kb) [file 12870_2018_1575_MOESM2_ESM.docx]
