# Supplementary material for: Construction of a highly saturated Genetic Map for Vitis by Next-generation Restriction Site-associated DNA Sequencing
Source: BMC Plant Biol. 2018 Dec 12;18:347. doi: 10.1186/s12870-018-1575-z (PMC6291968; doi:10.1186/s12870-018-1575-z)
Supplement: Supplementary file 3 — Figure S3. Heat map of the genetic linkage map. (ZIP 3270 kb) [file 12870_2018_1575_MOESM3_ESM.zip › instructions.docx]

**Each row and each column is a marker arranged in the order of the genetic map, each small square represents the recombination rate between two markers, and the change of color from purple to yellow represents the change of recombination rate from large to small.**
