# Supplementary figures and images for: Construction of a highly saturated Genetic Map for Vitis by Next-generation Restriction Site-associated DNA Sequencing
Source: BMC Plant Biol. 2018 Dec 12;18:347. doi: 10.1186/s12870-018-1575-z (PMC6291968; doi:10.1186/s12870-018-1575-z)

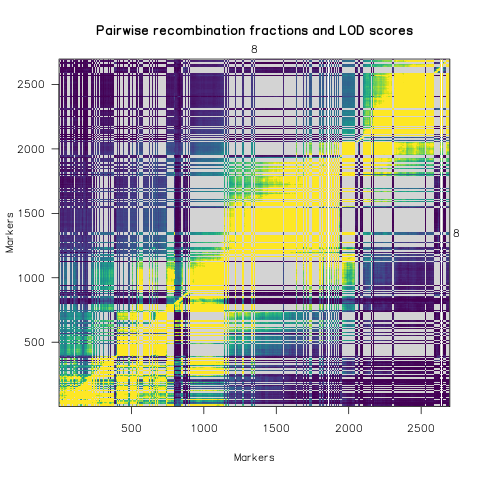

Supplement: Supplementary file 3 — Figure S3. Heat map of the genetic linkage map. (ZIP 3270 kb) [file 12870_2018_1575_MOESM3_ESM.zip › LG8.heatMap.sexAver.png]

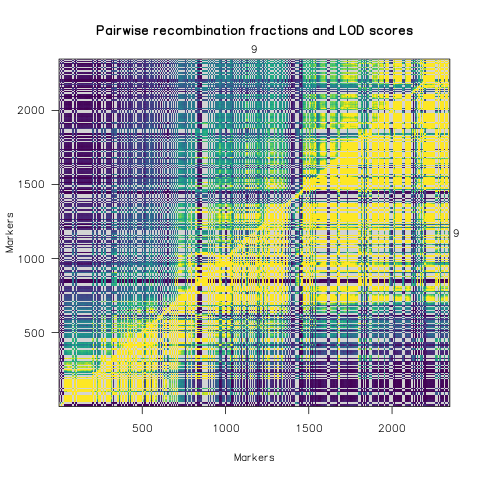

Supplement: Supplementary file 3 — Figure S3. Heat map of the genetic linkage map. (ZIP 3270 kb) [file 12870_2018_1575_MOESM3_ESM.zip › LG9.heatMap.sexAver.png]

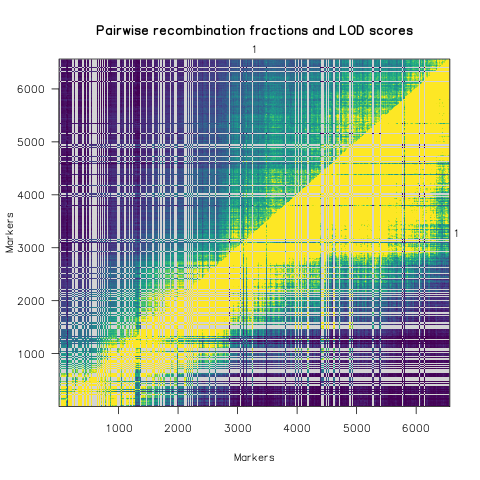

Supplement: Supplementary file 3 — Figure S3. Heat map of the genetic linkage map. (ZIP 3270 kb) [file 12870_2018_1575_MOESM3_ESM.zip › LG1.heatMap.sexAver.png]

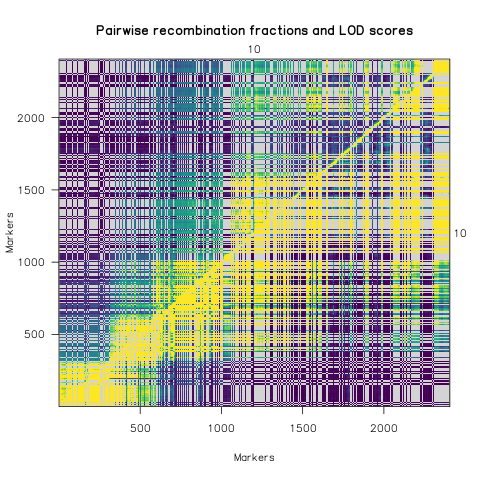

Supplement: Supplementary file 3 — Figure S3. Heat map of the genetic linkage map. (ZIP 3270 kb) [file 12870_2018_1575_MOESM3_ESM.zip › LG10.heatMap.sexAver.png]

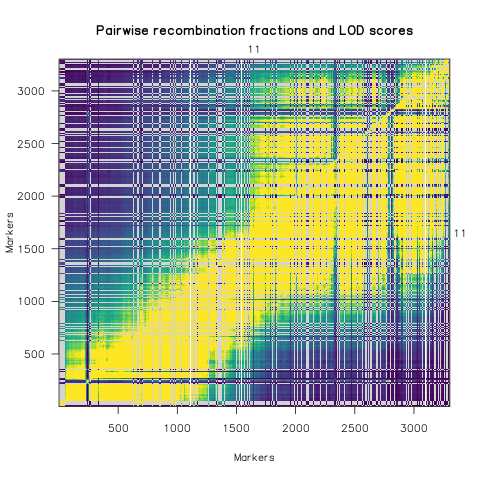

Supplement: Supplementary file 3 — Figure S3. Heat map of the genetic linkage map. (ZIP 3270 kb) [file 12870_2018_1575_MOESM3_ESM.zip › LG11.heatMap.sexAver.png]

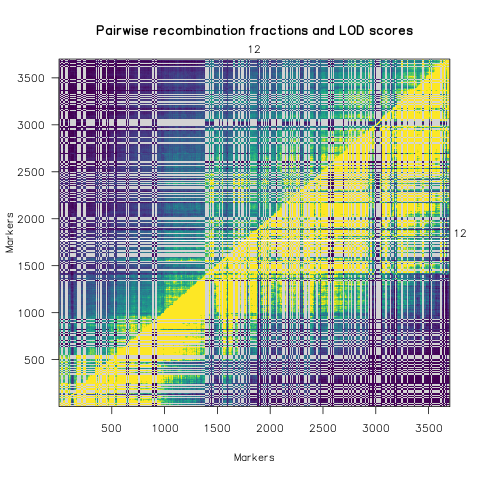

Supplement: Supplementary file 3 — Figure S3. Heat map of the genetic linkage map. (ZIP 3270 kb) [file 12870_2018_1575_MOESM3_ESM.zip › LG12.heatMap.sexAver.png]

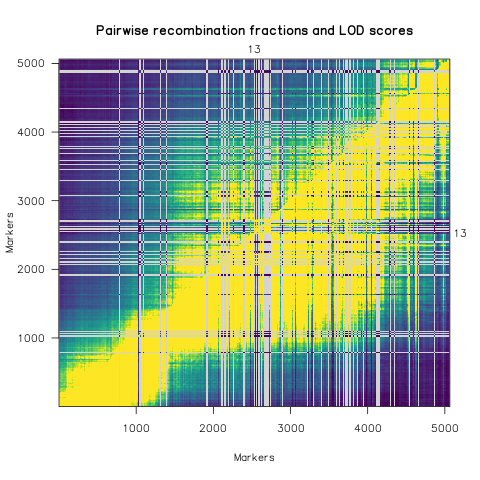

Supplement: Supplementary file 3 — Figure S3. Heat map of the genetic linkage map. (ZIP 3270 kb) [file 12870_2018_1575_MOESM3_ESM.zip › LG13.heatMap.sexAver.png]

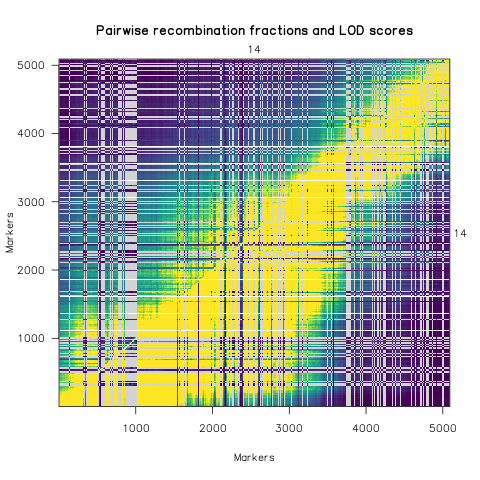

Supplement: Supplementary file 3 — Figure S3. Heat map of the genetic linkage map. (ZIP 3270 kb) [file 12870_2018_1575_MOESM3_ESM.zip › LG14.heatMap.sexAver.png]

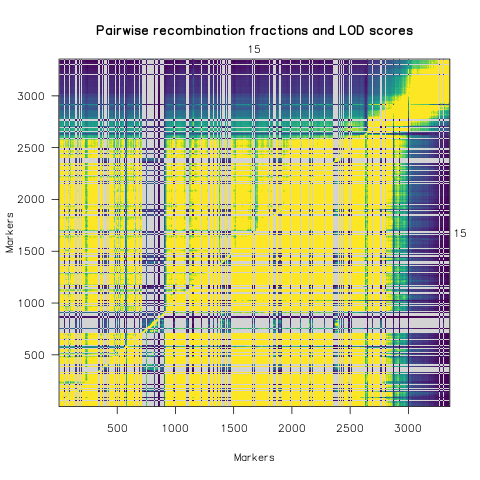

Supplement: Supplementary file 3 — Figure S3. Heat map of the genetic linkage map. (ZIP 3270 kb) [file 12870_2018_1575_MOESM3_ESM.zip › LG15.heatMap.sexAver.png]

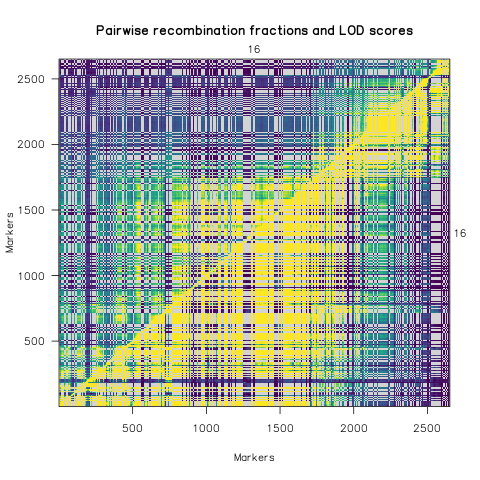

Supplement: Supplementary file 3 — Figure S3. Heat map of the genetic linkage map. (ZIP 3270 kb) [file 12870_2018_1575_MOESM3_ESM.zip › LG16.heatMap.sexAver.png]

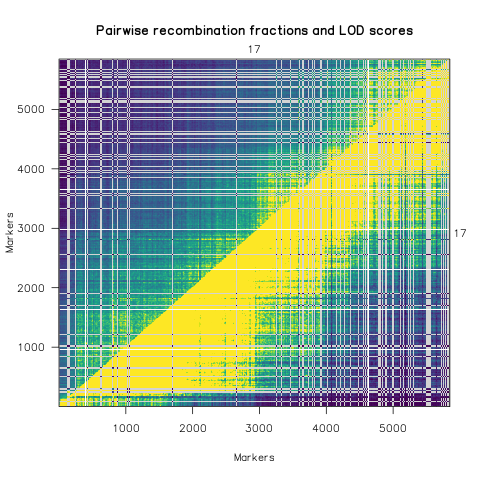

Supplement: Supplementary file 3 — Figure S3. Heat map of the genetic linkage map. (ZIP 3270 kb) [file 12870_2018_1575_MOESM3_ESM.zip › LG17.heatMap.sexAver.png]

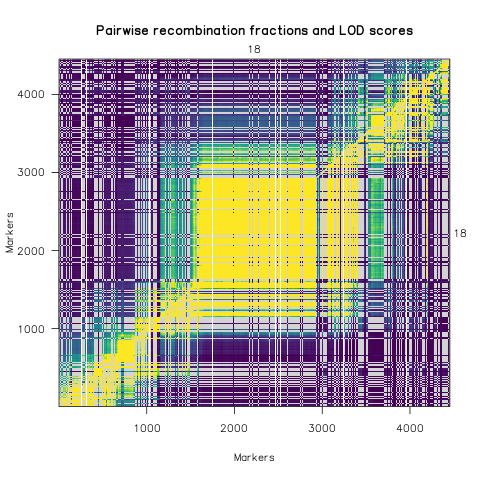

Supplement: Supplementary file 3 — Figure S3. Heat map of the genetic linkage map. (ZIP 3270 kb) [file 12870_2018_1575_MOESM3_ESM.zip › LG18.heatMap.sexAver.png]

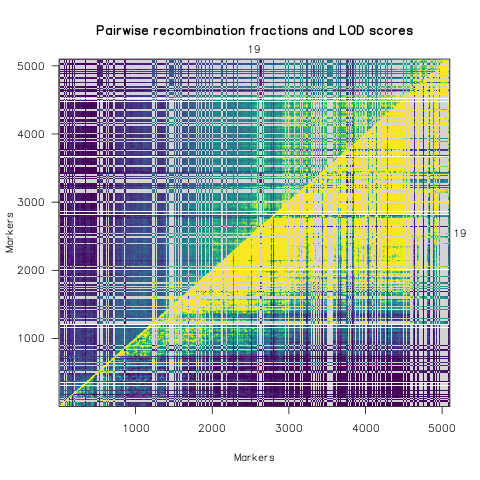

Supplement: Supplementary file 3 — Figure S3. Heat map of the genetic linkage map. (ZIP 3270 kb) [file 12870_2018_1575_MOESM3_ESM.zip › LG19.heatMap.sexAver.png]

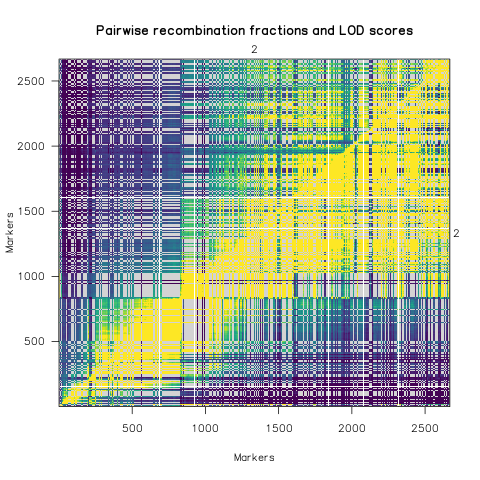

Supplement: Supplementary file 3 — Figure S3. Heat map of the genetic linkage map. (ZIP 3270 kb) [file 12870_2018_1575_MOESM3_ESM.zip › LG2.heatMap.sexAver.png]

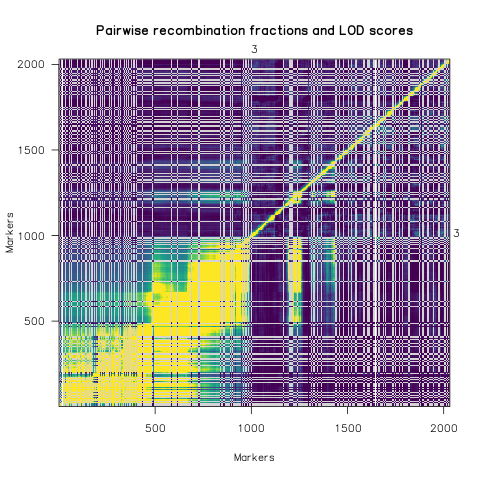

Supplement: Supplementary file 3 — Figure S3. Heat map of the genetic linkage map. (ZIP 3270 kb) [file 12870_2018_1575_MOESM3_ESM.zip › LG3.heatMap.sexAver.png]

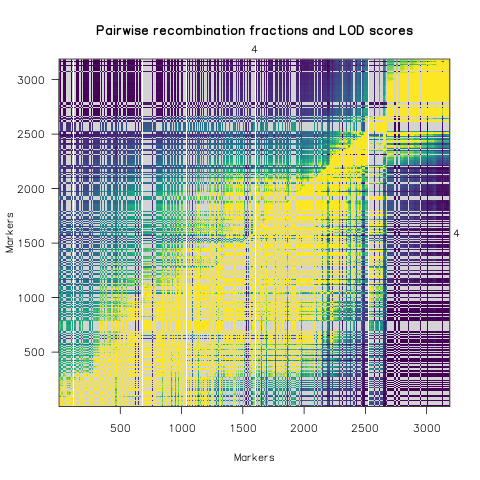

Supplement: Supplementary file 3 — Figure S3. Heat map of the genetic linkage map. (ZIP 3270 kb) [file 12870_2018_1575_MOESM3_ESM.zip › LG4.heatMap.sexAver.png]

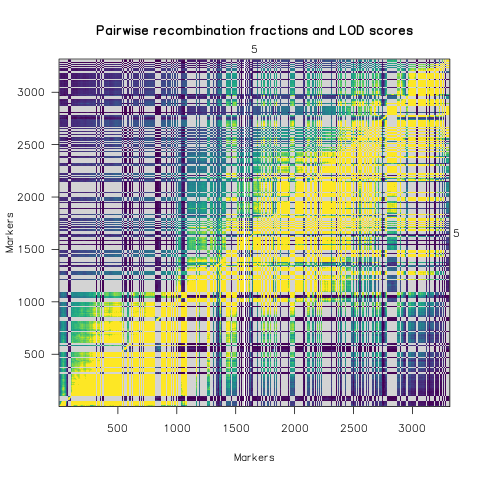

Supplement: Supplementary file 3 — Figure S3. Heat map of the genetic linkage map. (ZIP 3270 kb) [file 12870_2018_1575_MOESM3_ESM.zip › LG5.heatMap.sexAver.png]

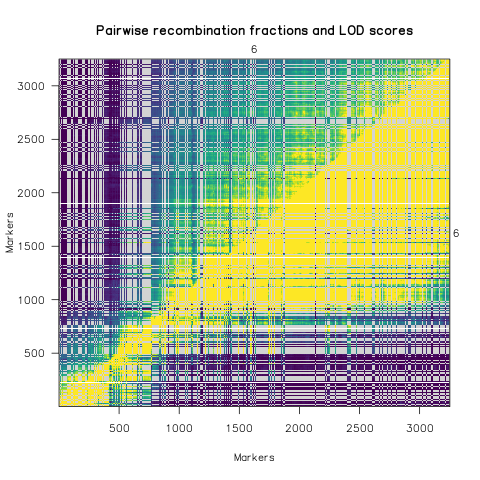

Supplement: Supplementary file 3 — Figure S3. Heat map of the genetic linkage map. (ZIP 3270 kb) [file 12870_2018_1575_MOESM3_ESM.zip › LG6.heatMap.sexAver.png]

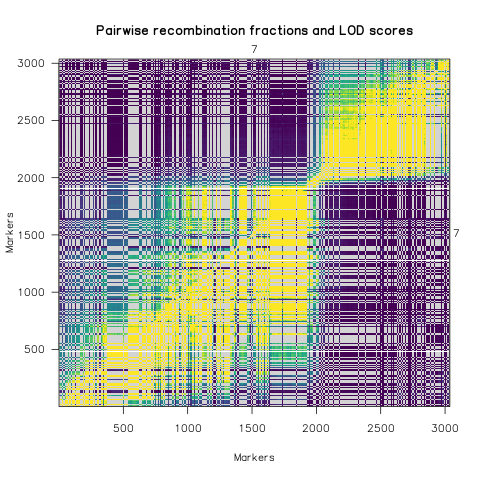

Supplement: Supplementary file 3 — Figure S3. Heat map of the genetic linkage map. (ZIP 3270 kb) [file 12870_2018_1575_MOESM3_ESM.zip › LG7.heatMap.sexAver.png]
